# Supplementary material for: The N-terminal region of malaria vaccine candidate asparagine-rich merozoite antigen is immunodominant and targeted by polyreactive antibodies
Source: Dis Model Mech. 2026 Jun 2;19(5):dmm052979. doi: 10.1242/dmm.052979 (PMC13312920; doi:10.1242/dmm.052979)
Supplement: Supplementary information [file dmm-19-052979-s1.pdf]

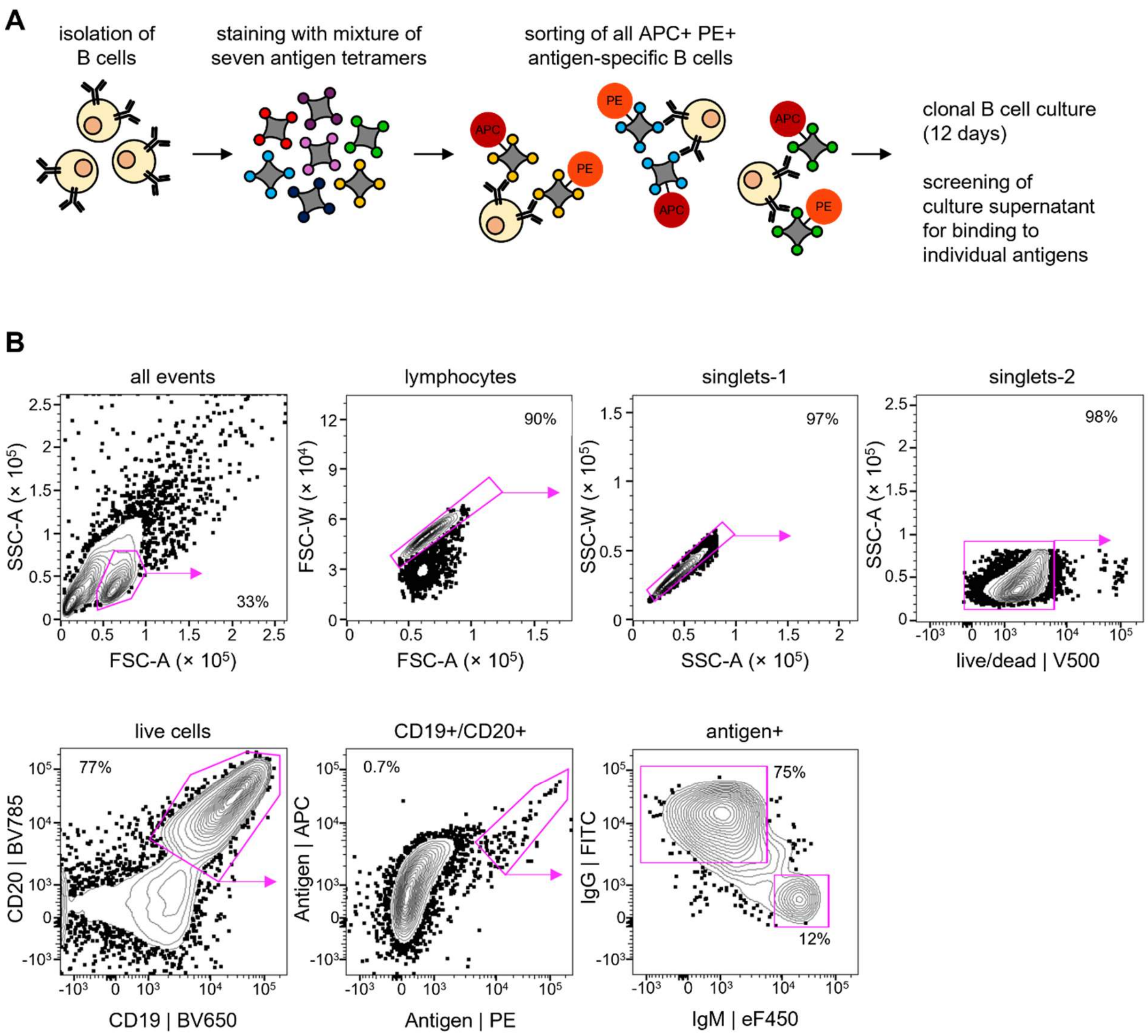

Fig. S1. Isolation strategy of antigen-specific IgM⁺ and IgG⁺ B cells. A) Schematic overview of the isolation of antigen-specific B cells using tetramers. B cells were isolated by negative selection and were subsequently stained with a mixture of antigen tetramers for seven *P. falciparum* antigens (PfMSP1, PfMSP3, PfAMA1, PfVFT, Pf41, Pf113, and PfARMA). For each antigen, two versions of the same tetramer were produced, one conjugated to PE, the other to APC. All $PE^{+}APC^{+}$ B cells were sorted at a density of one cell per well and were subsequently cultured to allow differentiation into antibody-secreting cells. Culture supernatants were screened for antibody reactivity to individual antigens by Luminex assay. **B)** Gating strategy. Live, single lymphocytes were gated on CD19 and CD20 expression, followed by selection of $PE^{+}APC^{+}$ B cells. Finally, antigen-tetramer^{+} cells were sorted into two populations based on IgM or IgG expression.

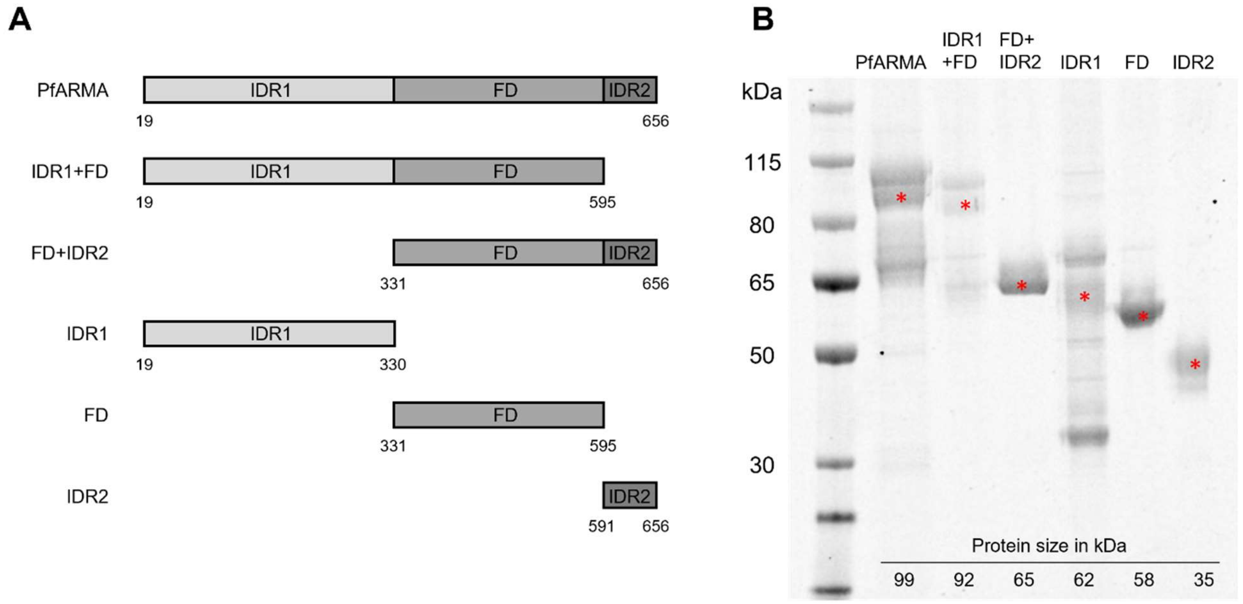

Fig. S2. Production of recombinant ARMA protein fragments. A) Overview of recombinant full-length PfARMA and fragments of PfARMA with start and end amino acid residues indicated. **B)** Gel electrophoresis analysis of recombinant PfARMA proteins after purification. The red asterisks indicate the band corresponding to the expected protein size as indicated in the bottom. IDR, intrinsically disordered region; FD, folded domain.

Disease Models & Mechanisms • Supplementary information

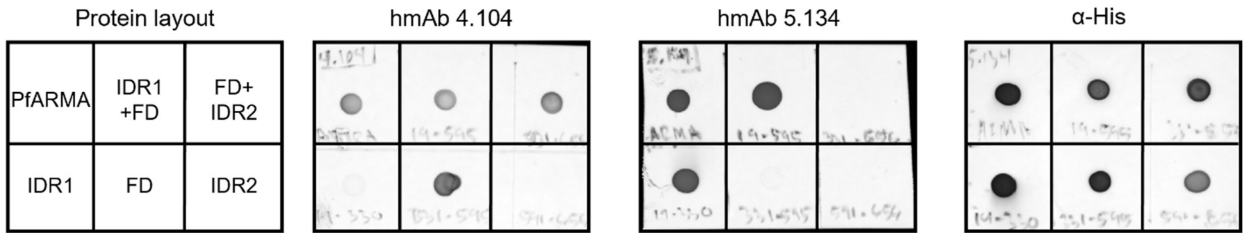

Fig. S3. Reactivity of recombinant human monoclonal antibodies to PfARMA. Dot-blot analysis showing reactivity of hmAbs 4.104 and 5.134 to full-length PfARMA and fragments of PfARMA. As a positive control for the presence of protein, a third dot-blot was stained in parallel with an anti-His antibody (right).

Disease Models & Mechanisms • Supplementary information

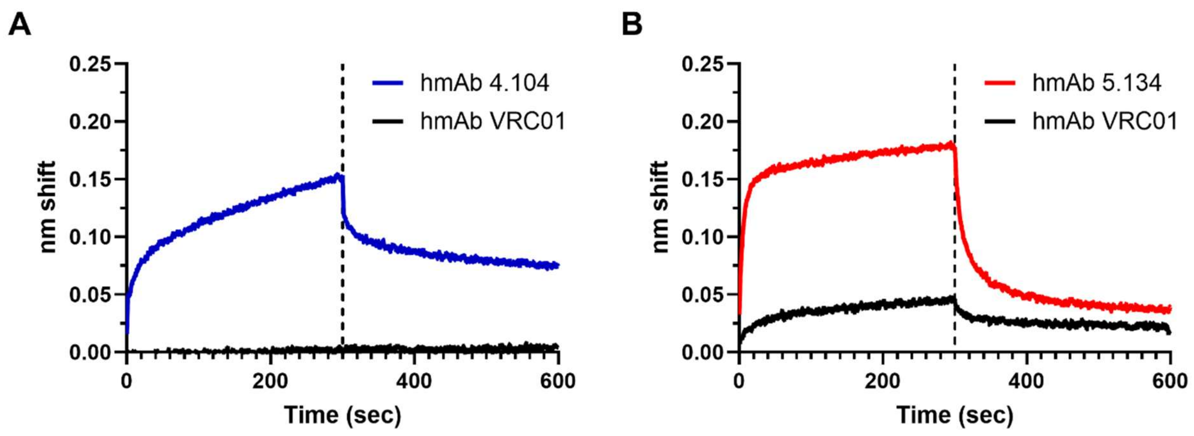

Fig. S4. Biolayer interferometry data of hmAbs binding to ARMA. A) hmAb 4.104 binding to ARMA FD (residues 331-595). **B)** hmAb 5.134 binding to ARMA IDR1 (residues 220-269) fused to maltose binding protein. hmAb VRC01 is an anti-HIV antibody used as a negative control.

Disease Models & Mechanisms • Supplementary information

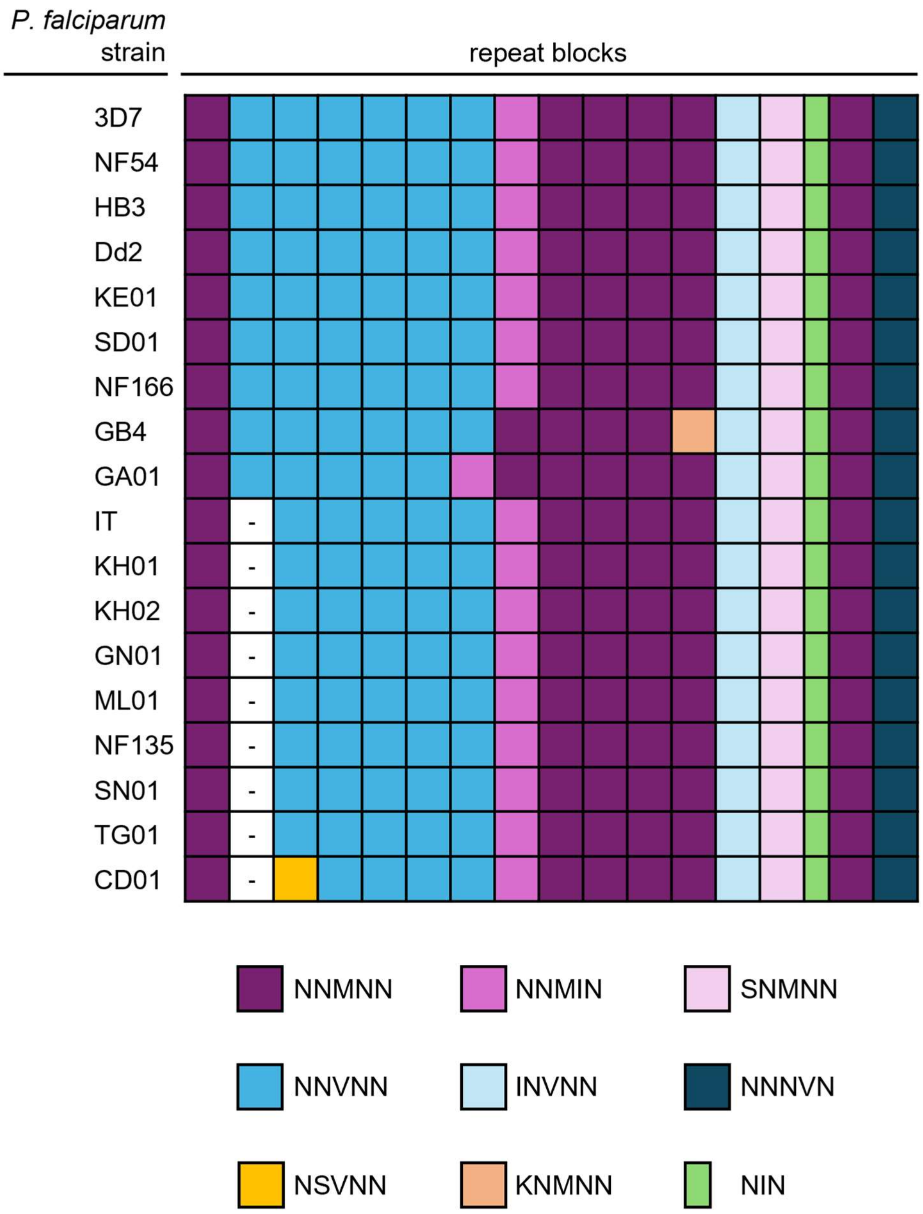

Fig. S5. Schematic protein alignment of the PfARMA repeat region. For 18 *P. falciparum* strains available from PlasmoDB, each 5-amino acid repeat is shown as a square and color-coded by amino acid sequence.

Disease Models & Mechanisms • Supplementary information

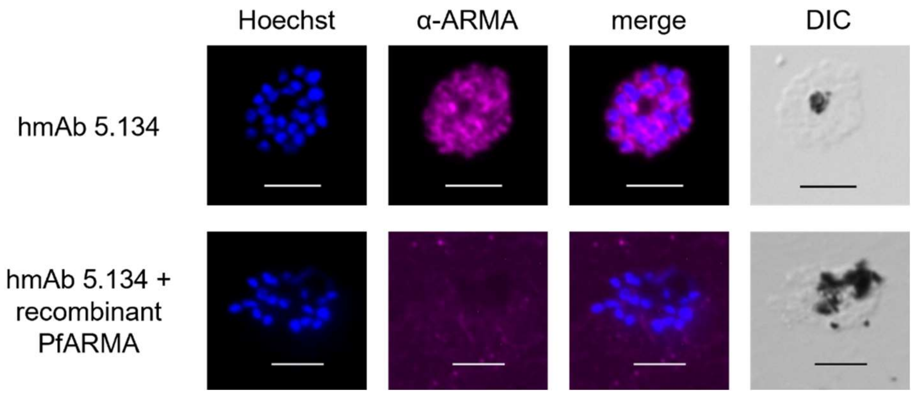

Fig. S6. Immunoblocking of hmAb 5.134 in immunofluorescence assays.
Immunofluorescence images of segmented schizonts stained with hmAb 5.134 against PfARMA (top) and the same experiment that included the addition of free recombinant PfARMA during primary antibody incubation (bottom). Scale bar denotes 5 µm.

Disease Models & Mechanisms • Supplementary information

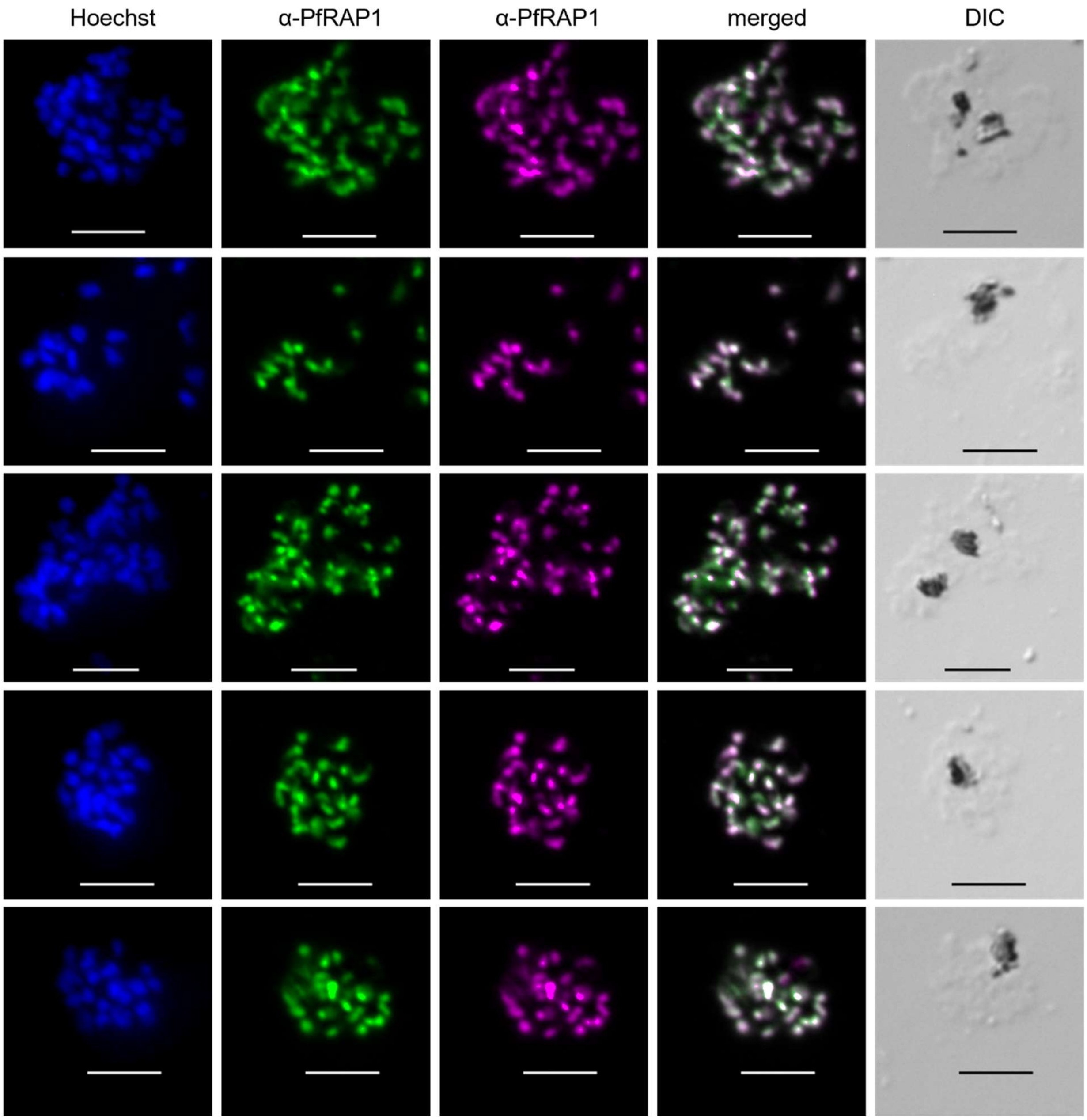

Fig. S7. Immunofluorescence images of segmented schizonts. Parasites were stained with Hoechst 33342 (DNA), an antibody against rhoptry protein PfRAP1 and two different secondary antibodies to serve as a positive control for colocalization studies. Scale bar denotes 5 µm.

Disease Models & Mechanisms • Supplementary information

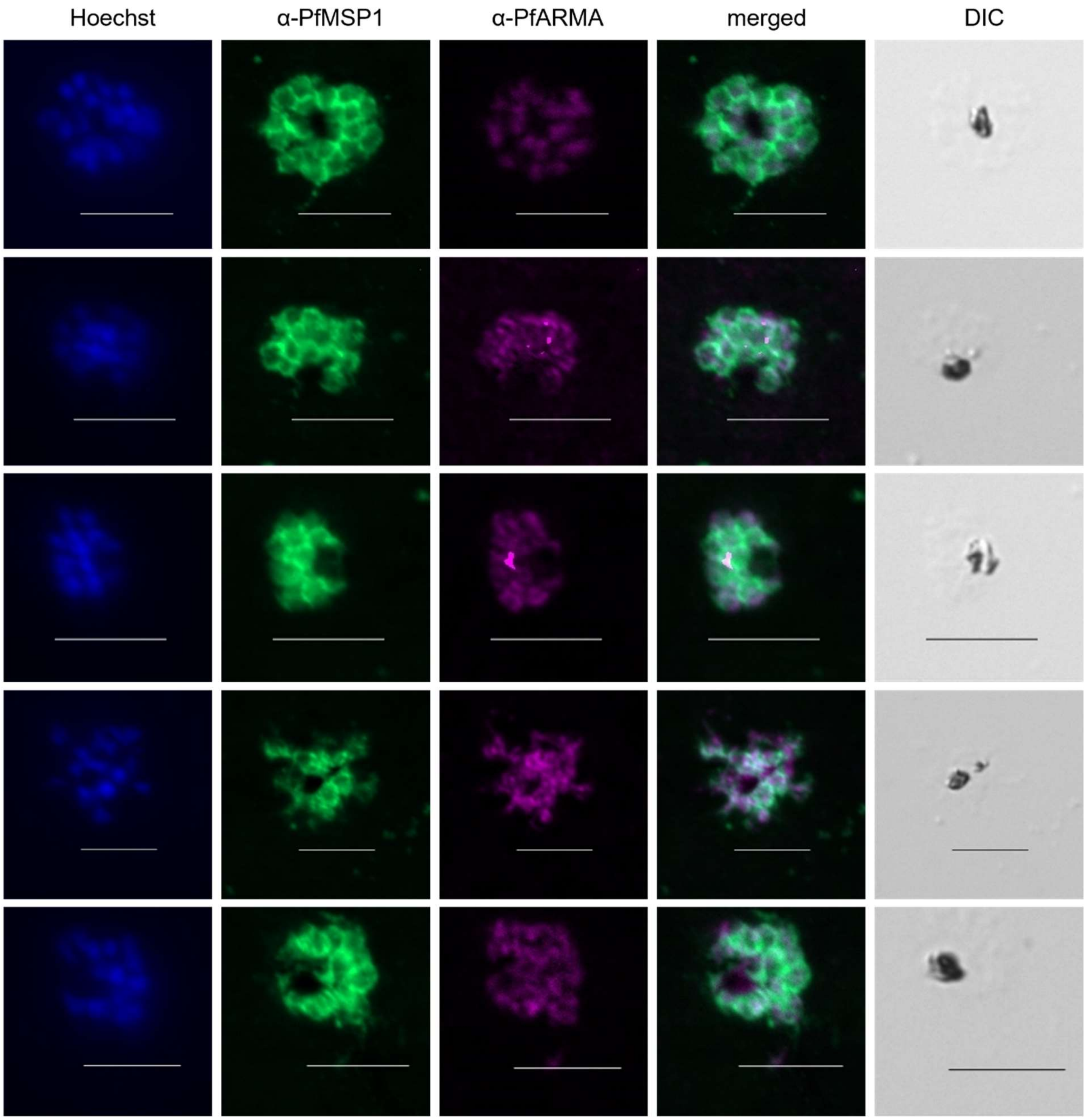

Fig. S8. Immunofluorescence images of segmented schizonts. Parasites were stained with Hoechst 33342 (DNA), hmAb 5.134 against PfARMA and an antibody against merozoite surface protein PfMSP1. Scale bar denotes 5 µm.

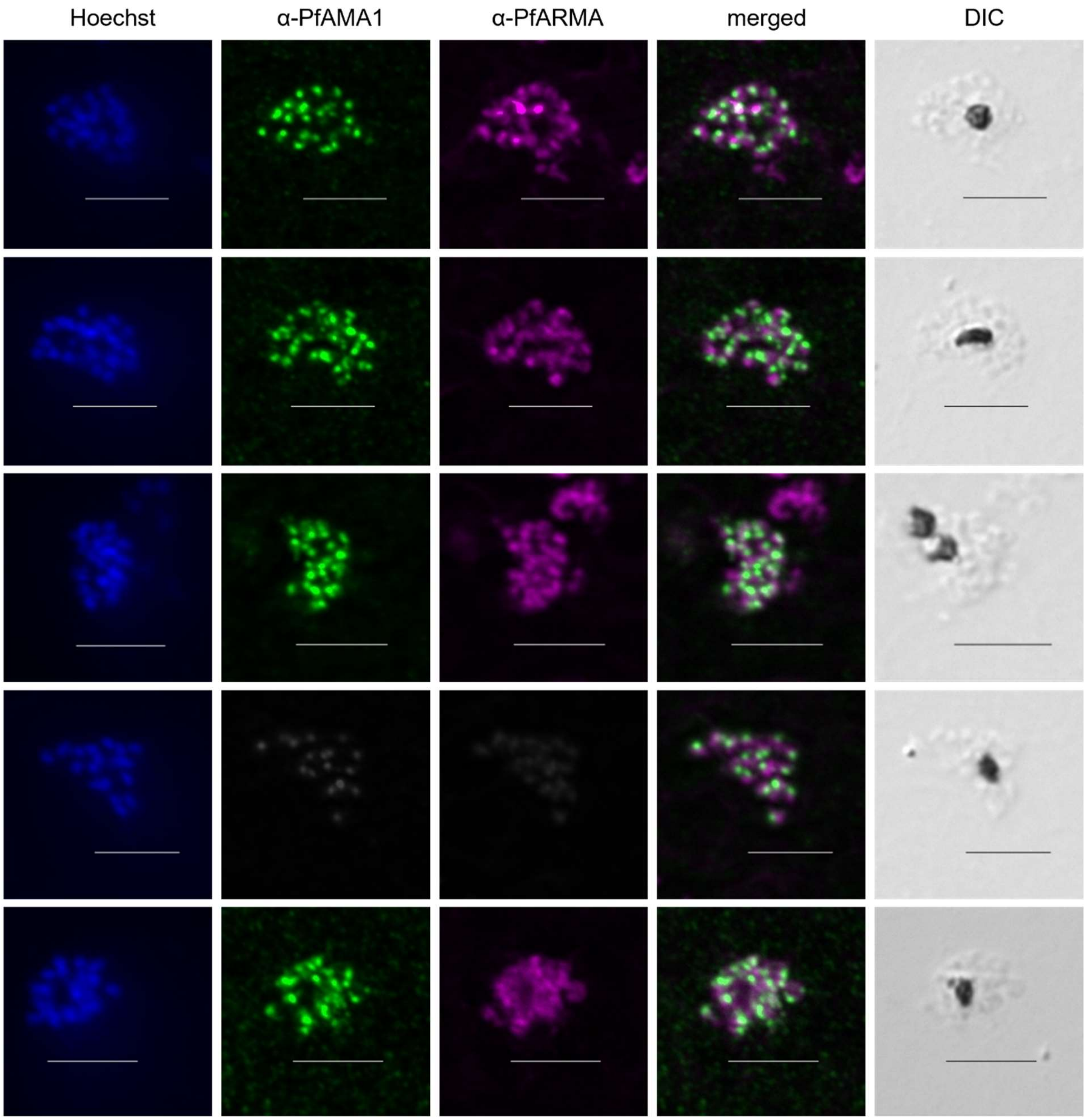

Fig. S9. Immunofluorescence images of segmented schizonts. Parasites were stained with Hoechst 33342 (DNA), hmAb 5.134 against PfARMA and an antibody against microneme protein PfAMA1. Scale bar denotes 5 μm.

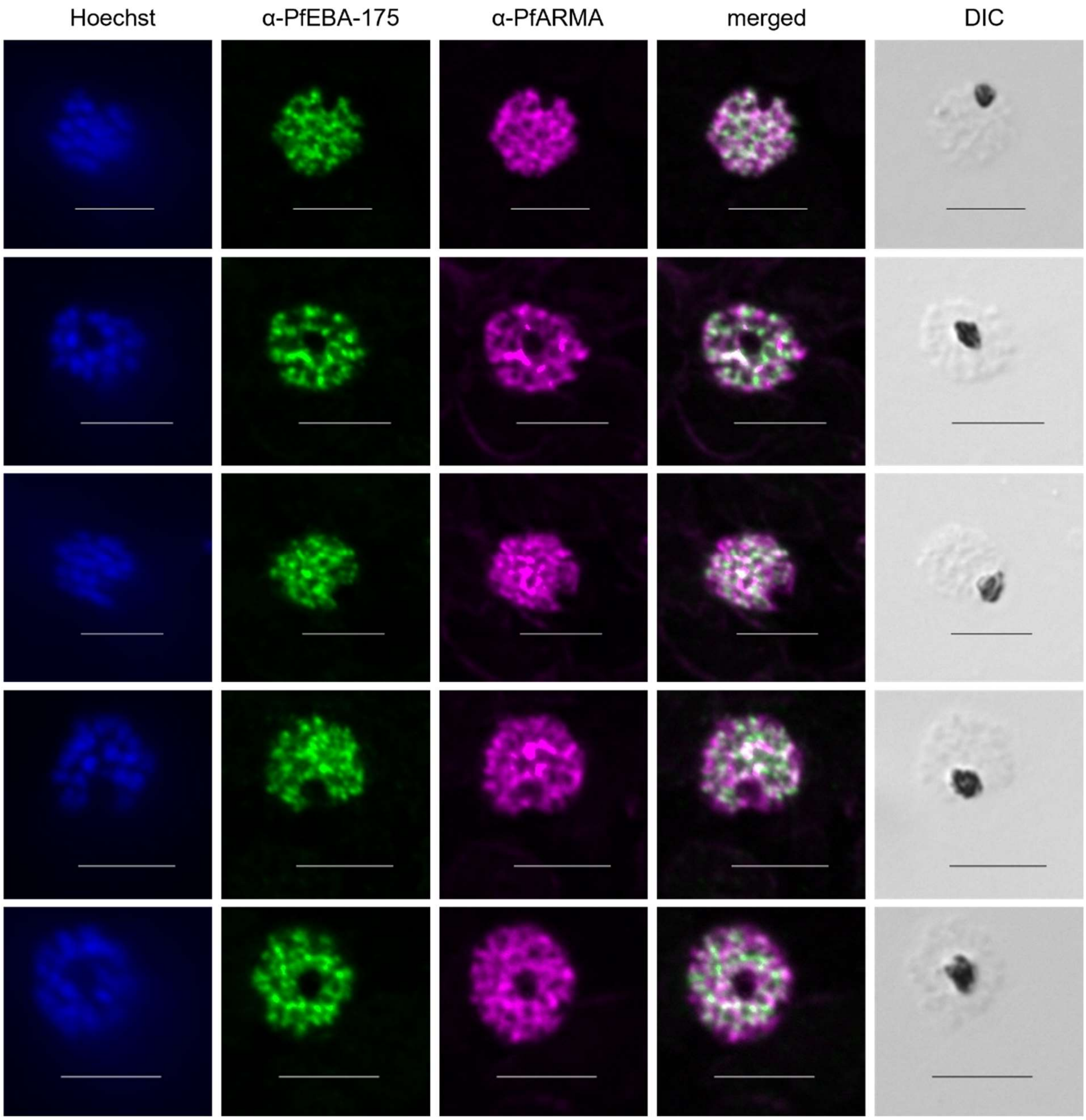

Fig. S10. Immunofluorescence images of segmented schizonts. Parasites were stained with Hoechst 33342 (DNA), hmAb 5.134 against PfARMA and an antibody against microneme protein PfEBA-175. Scale bar denotes 5 µm.

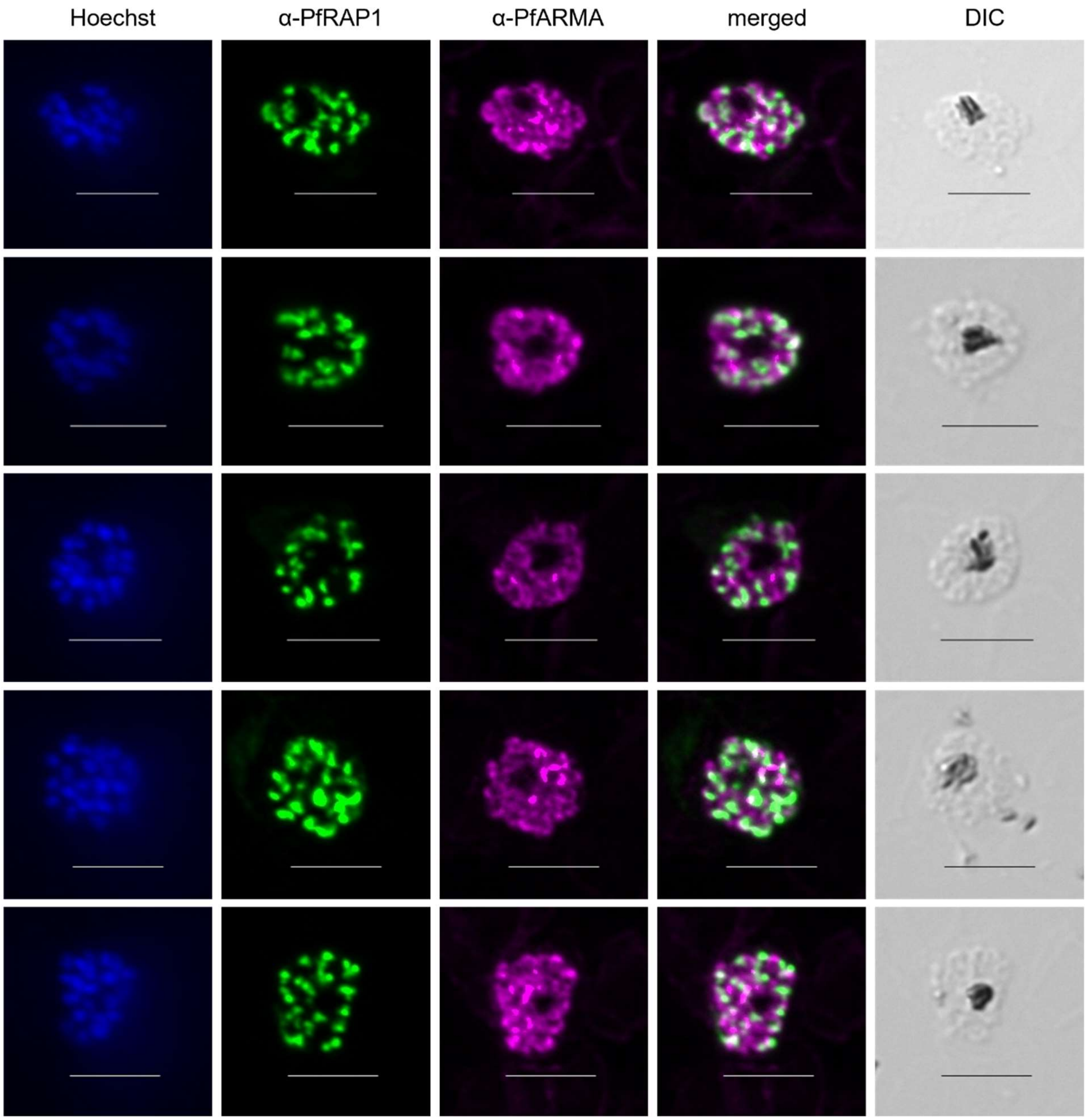

Fig. S11. Immunofluorescence images of segmented schizonts. Parasites were stained with Hoechst 33342 (DNA), hmAb 5.134 against PfARMA and an antibody against rhoptry protein PfRAP1. Scale bar denotes 5 µm.

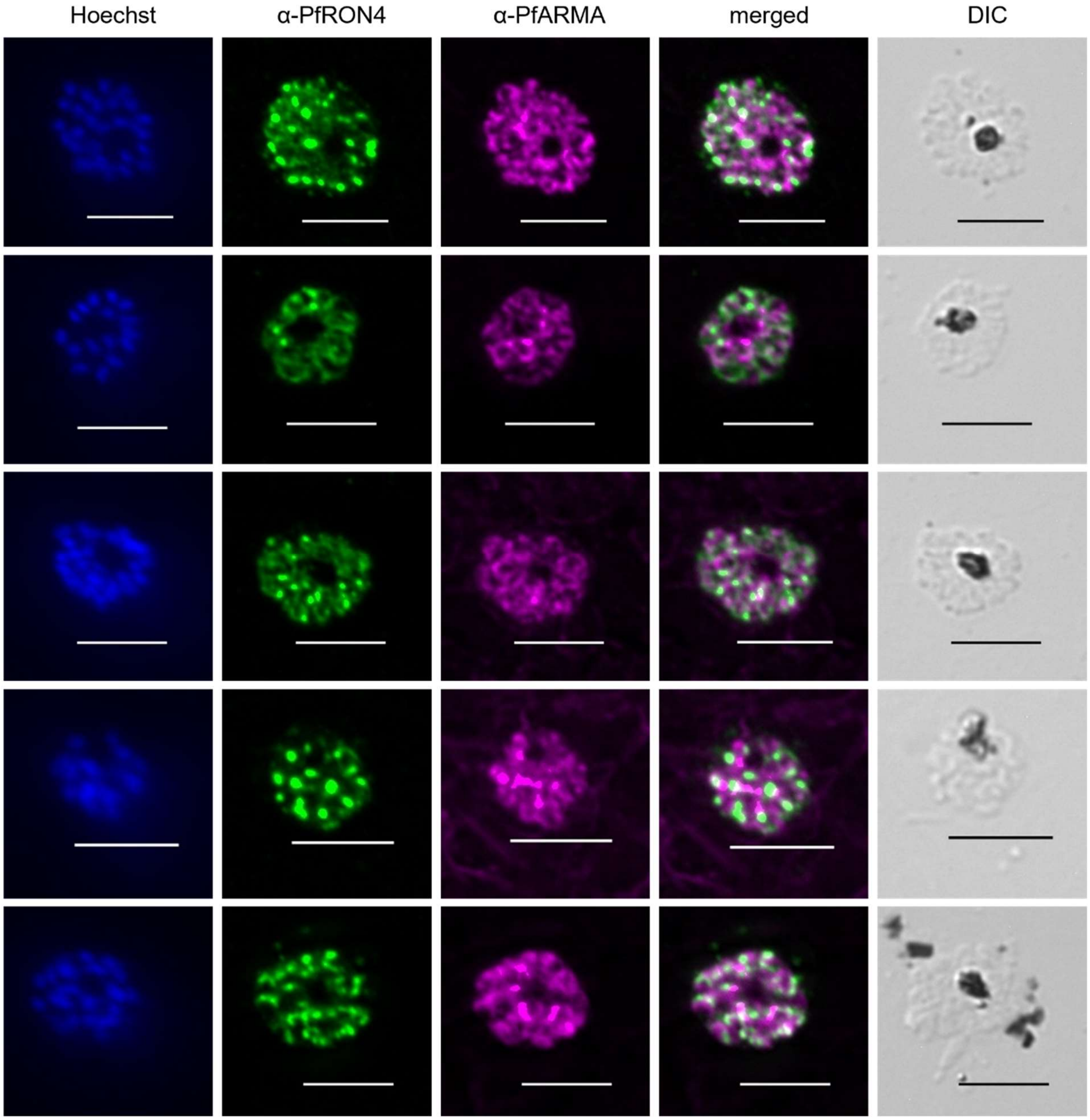

Fig. S12. Immunofluorescence images of segmented schizonts. Parasites were stained with Hoechst 33342 (DNA), hmAb 5.134 against PfARMA and an antibody against rhoptry protein PfRON4. Scale bar denotes 5 µm.

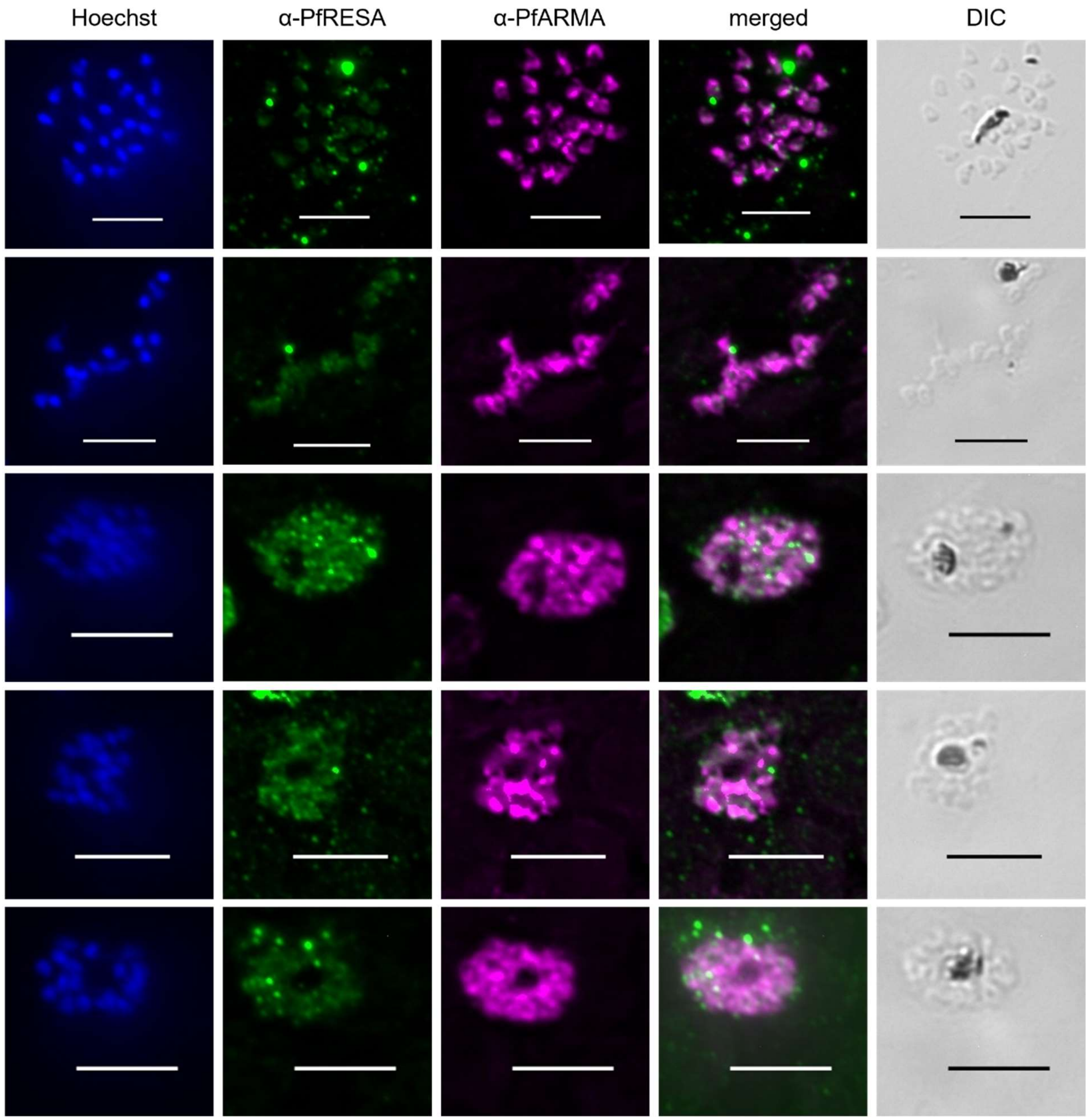

Fig. S13. Immunofluorescence images of segmented schizonts. Parasites were stained with Hoechst 33342 (DNA), hmAb 5.134 against PfARMA and an antibody against dense granule protein PfRESA. Scale bar denotes 5 µm.

Disease Models & Mechanisms • Supplementary information

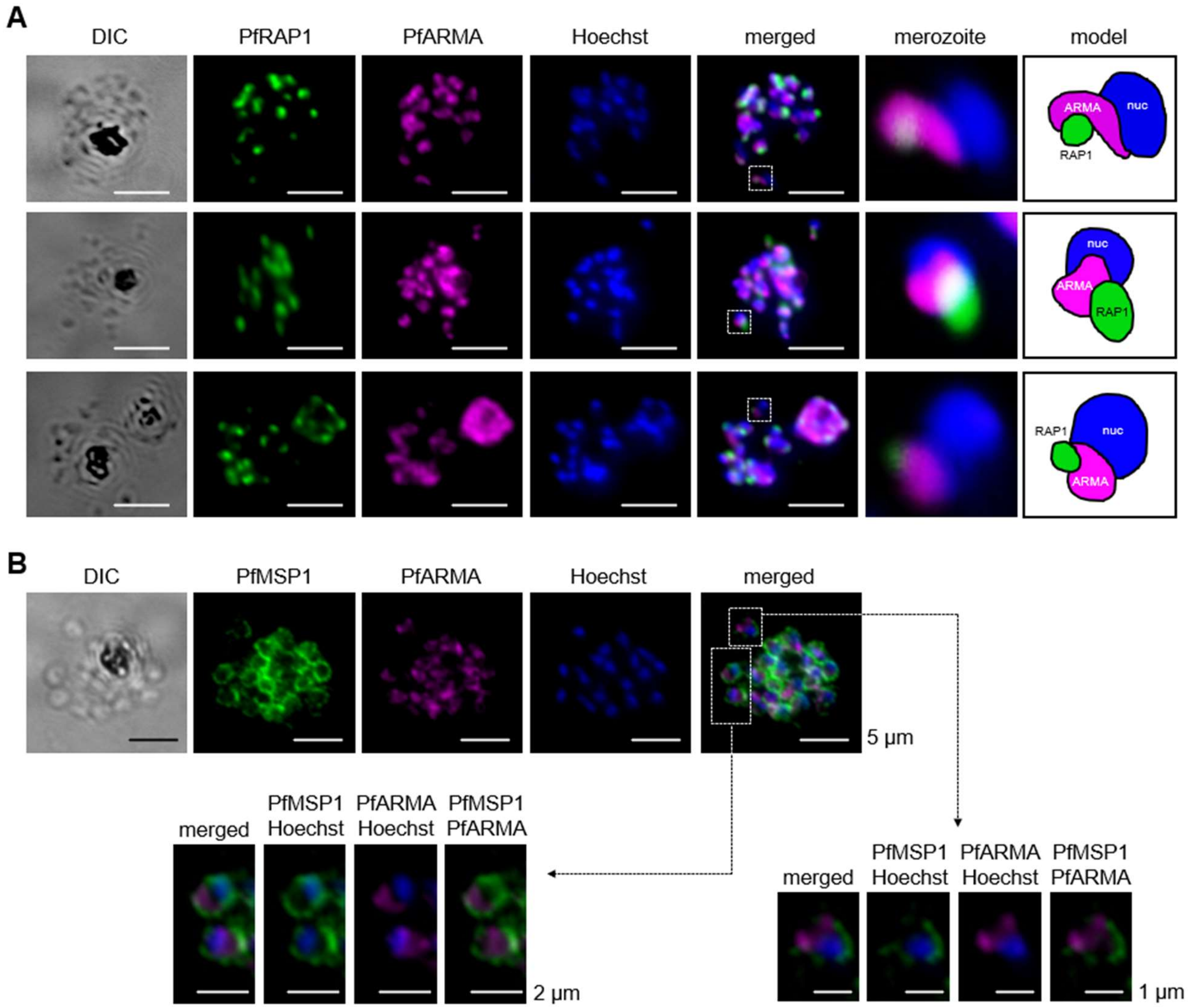

Fig. S14. Immunofluorescence images of merozoites released from a fully segmented schizont. A) Merozoites stained with Hoechst 33342 (DNA), hmAb 5.134 against PfARMA, and an antibody against rhoptry protein PfRAP1. Individual merozoites highlighted in the merged image are enlarged and a model depicting the relative localization of nucleus (nuc), PfARMA, and PfRAP1 is shown on the left. The scale bar denotes 5 µm. **B)** Merozoites stained with Hoechst 33342 (DNA), hmAb 5.134 against PfARMA, and an antibody against merozoite surface protein PfMSP1. Merozoites highlighted in the merged image are enlarged. The size of the scale bar is denoted for each set of images. Images are representative of 48 and 62 parasites analyzed in two independent PfRAP1 and PfMSP1 colocalization experiments, respectively.

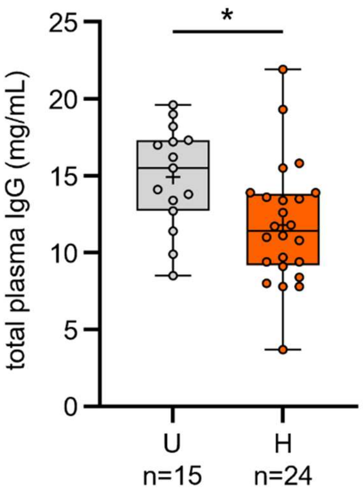

Fig. S15. Total IgG concentrations in plasma. IgG concentrations in plasma samples from *P. falciparum*-unexposed individuals (U) and *P. falciparum*-exposed adults with high levels of immunity to malaria (H) were determined using ELISA. Center line, median; box limits, upper and lower quartiles; whiskers, min/max values; +, mean. The difference between groups was tested using an unpaired student's t-test. * $P < 0.05$.

PfARMA IDR1

```
  1 MNLNKISVLLKIIIFKNTLYIFDNVCTSIHVNDLGYKVSDTINSFKYKKNYDNSVVLDKV
 61 PDTPLDNNAKLSFQELLRKRSSDDDDDNDDEGDNDDDDDDNDNNNNNNNNNNNNMNRFYNM
121 ASIQPAAFNNISQNGNQSFTARMKQNLSKYNPFKKSGSNNNGKVNNTNENVDEDNNDNVD
181 DNNTKKKHRHIKHKSQGTLSNDGSNNMNNNNVNNNNVNNNNVNNNNVNNNNVNNNNVNNN
241 NMINNNMNNNNMNNNNMNNNNMNNINVNNSNMNNNINNNMNNNNNVNASNILLGASALTG
301 AAISGQNQNGINNNQNVVNNNTNNGTIQNS
```

Negatively charged patch 1
20 amino acid residues
16 negatively charged residues
Net charge per residue: -0.80

DDDDDNDDEGDNDDDDDDND

Negatively charged patch 2
13 amino acid residues
7 negatively charged residues
Net charge per residue: -0.53

ENVDEDNNDNVDD

Asparagine-rich repeat region
83 amino acid residues
63 asparagine residues (shown in white)

NNMNNNNVNNNNVNNNNVNNNNVNNNNVNNNNVNNNNMINNNMNNNNMNNNNMNNNNMNNINVNNSNMNNNINNNMNNNNNVN

Fig. S16. Sequence characteristics of PfARMA IDR1. The sequence shown is from the *P. falciparum* reference strain 3D7.

Table S1. Characteristics of donors included in this study.

Available for download at
https://journals.biologists.com/dmm/article-lookup/doi/10.1242/dmm.052979#supplementary-data

Table S2. Number and percentage of human monoclonal antibodies derived from IgM+ and IgG+ B cells by merozoite antigen.

Available for download at
https://journals.biologists.com/dmm/article-lookup/doi/10.1242/dmm.052979#supplementary-data

Disease Models & Mechanisms • Supplementary information

Table S3. Reactivity of clonal B cell supernatants against the three regions of PfARMA.

Available for download at
https://journals.biologists.com/dmm/article-lookup/doi/10.1242/dmm.052979#supplementary-data

Table S4. Growth-inhibitory activity of hmAbs 4.104 and 5.134.

Available for download at
https://journals.biologists.com/dmm/article-lookup/doi/10.1242/dmm.052979#supplementary-data

Table S5. *P. falciparum* strain 3D7 peptides bound by hmAb 5.134.

Available for download at
https://journals.biologists.com/dmm/article-lookup/doi/10.1242/dmm.052979#supplementary-data

Table S6. Colocalization metrics for PfARMA and various merozoite antigens.

Available for download at
https://journals.biologists.com/dmm/article-lookup/doi/10.1242/dmm.052979#supplementary-data

Table S7. Characteristics of donor groups with differing levels of immunity to *P. falciparum* malaria.

Available for download at
https://journals.biologists.com/dmm/article-lookup/doi/10.1242/dmm.052979#supplementary-data

Table S8. Plasma IgM and IgG reactivity to PfMSP1, PfARMA, and PfARMA fragments.

Available for download at
https://journals.biologists.com/dmm/article-lookup/doi/10.1242/dmm.052979#supplementary-data

Table S9. Plasma IgG reactivity to *P. falciparum* antigens among *P. falciparum*-naïve individuals from the USA.

Available for download at
https://journals.biologists.com/dmm/article-lookup/doi/10.1242/dmm.052979#supplementary-data

Table S10. Reactivity of IgG purified from plasma of a *P. falciparum*-naïve US donor to PfARMA and PfMSP1.

Available for download at
https://journals.biologists.com/dmm/article-lookup/doi/10.1242/dmm.052979#supplementary-data

Table S11. Total plasma IgG concentrations and PfARMA IgG reactivity in *P. falciparum*-naïve U.S. donors and *P. falciparum*-exposed Ugandan individuals.

Available for download at
https://journals.biologists.com/dmm/article-lookup/doi/10.1242/dmm.052979#supplementary-data

Table S12. Reactivity of recombinant IgG subclasses with specificity for an unrelated *P. falciparum* antigen to PfARMA and PfMSP1.

Available for download at
https://journals.biologists.com/dmm/article-lookup/doi/10.1242/dmm.052979#supplementary-data

Table S13. Reactivity of plasma IgG, affinity-purified autoantibodies, and flowthrough fraction to PfARMA and six other merozoite antigens.

Available for download at
https://journals.biologists.com/dmm/article-lookup/doi/10.1242/dmm.052979#supplementary-data

Table S14. Results from polyreactivity ELISAs.

Available for download at
https://journals.biologists.com/dmm/article-lookup/doi/10.1242/dmm.052979#supplementary-data

Table S15. *P. falciparum* strain 3D7 peptides bound by plasma from *P. falciparum*-naïve US donors and *P. falciparum*-exposed Ugandan individuals.

Available for download at
https://journals.biologists.com/dmm/article-lookup/doi/10.1242/dmm.052979#supplementary-data

Table S16. Oligos used for amplification and cloning of PfARMA fragments and hmAbs.

Available for download at
https://journals.biologists.com/dmm/article-lookup/doi/10.1242/dmm.052979#supplementary-data

Table S17. Oligos used for cDNA synthesis and amplification of antibody heavy and light chain variable regions.

Available for download at
https://journals.biologists.com/dmm/article-lookup/doi/10.1242/dmm.052979#supplementary-data

Disease Models & Mechanisms • Supplementary information
